# Transcriptome and Proteome Response of *Rhipicephalus annulatus* Tick Vector to *Babesia bigemina* Infection

**DOI:** 10.3389/fphys.2019.00318

**Published:** 2019-04-02

**Authors:** Sandra Antunes, Joana Couto, Joana Ferrolho, Gustavo Seron Sanches, José Octavio Merino Charrez, Ned De la Cruz Hernández, Monica Mazuz, Margarita Villar, Varda Shkap, José de la Fuente, Ana Domingos

**Affiliations:** ^1^Global Health and Tropical Medicine, Instituto de Higiene e Medicina Tropical, Universidade Nova de Lisboa, Lisbon, Portugal; ^2^Facultad de Medicina Veterinaria y Zootecnia, Universidad Autónoma de Tamaulipas, Ciudad Victoria, Mexico; ^3^Kimron Veterinary Institute, Bet Dagan, Israel; ^4^SaBio, Instituto de Investigación en Recursos Cinegéticos, IREC, CSIC-UCLM-JCCM, Ciudad Real, Spain; ^5^Department of Veterinary Pathobiology, Center for Veterinary Health Sciences, Oklahoma State University, Stillwater, OK, United States

**Keywords:** proteomics, transcriptomics, *Rhipicephalus annulatus*, *Babesia bigemina*, vector–pathogen interactions, apoptosis, stress response

## Abstract

A system biology approach was used to gain insight into tick biology and interactions between vector and pathogen. *Rhipicephalus annulatus* is one of the main vectors of *Babesia bigemina* which has a massive impact on animal health. It is vital to obtain more information about this relationship, to better understand tick and pathogen biology, pathogen transmission dynamics, and new potential control approaches. In ticks, salivary glands (SGs) play a key role during pathogen infection and transmission. RNA sequencing obtained from uninfected and *B. bigemina* infected SGs obtained from fed female ticks resulted in 6823 and 6475 unigenes, respectively. From these, 360 unigenes were found to be differentially expressed (*p* < 0.05). Reversed phase liquid chromatography–mass spectrometry identified a total of 3679 tick proteins. Among them 406 were differently represented in response to *Babesia* infection. The omics data obtained suggested that *Babesia* infection lead to a reduction in the levels of mRNA and proteins (*n* = 237 transcripts, *n* = 212 proteins) when compared to uninfected controls. Integrated transcriptomics and proteomics datasets suggested a key role for stress response and apoptosis pathways in response to infection. Thus, six genes coding for GP80, death-associated protein kinase (DAPK-1), bax inhibitor-1 related (BI-1), heat shock protein (HSP), heat shock transcription factor (PHSTF), and queuine trna-ribosyltransferase (QtRibosyl) were selected and RNA interference (RNAi) performed. Gene silencing was obtained for all genes except *phstf.* Knockdown of *gp80*, *dapk-1*, and *bi-1* led to a significant increase in *Babesia* infection levels while *hsp* and *QtRibosyl* knockdown resulted in a non-significant decrease of infection levels when compared to the respective controls. Gene knockdown did not affect tick survival, but engorged female weight and egg production were affected in the *gp80*, *dapk-1*, and *QtRibosyl*-silenced groups in comparison to controls. These results advanced our understanding of tick–*Babesia* molecular interactions, and suggested new tick antigens as putative targets for vaccination to control tick infestations and pathogen infection/transmission.

## Introduction

Babesiosis is a worldwide tick-borne hemoprotozoa disease caused by intra-erythrocytic parasites of the genus *Babesia* (Beugnet and Moreau, 2015; Lempereur et al., 2017). The disease may range from asymptomatic carrier to more severe states, characterized by hemolytic anemia, fever, hemoglobinuria, and occasionally death, affecting a large variety of mammals, including pets, farm, and wild animals and also humans (Schnittger et al., 2012). Human infection is mainly due to *Babesia microti* or *Babesia divergens*, and although it rarely occurs, it has been considered an emerging zoonosis due to the growing number of fatal cases (Leiby, 2006; Schnittger et al., 2012). In contrast, cattle babesiosis, caused by either *Babesia bovis* or *B. bigemina*, is an important disease causing high morbidity and mortality, thus leading to severe economic losses to the cattle industry (Chauvin et al., 2009). The tick species *R. annulatus* and *Rhipicephalus microplus*, the most important ticks of cattle in tropical and subtropical regions, conduce a negative impact on meat, milk, and leather productions and are considered as the main vectors and reservoirs of *B. bovis* and *B. bigemina* (Bock et al., 2004).

The application of chemical acaricides is the method of choice for tick control. However, it results in environmental contamination, selection of resistant ticks, and presence of residues in meat and milk, potentially harmful for animals and humans (Corson et al., 2001; Ghosh et al., 2007). To reduce these negative impacts, much attention has been directed to the development of new approaches that are efficient and at the same time environmentally friendly. Vaccination, as a prophylactic measure, stands out representing a promising and sustainable alternative for the control of ticks and tick-borne pathogens (Almazan et al., 2018). A vaccine targeting both tick fitness and pathogen competence is an attractive choice requiring the identification of tick molecules with a dual effect.

Several studies, based on omics approaches, have been conducted to understand the tick–pathogen interactions, identifying possible new vaccine candidates (Villar et al., 2017). System biology approaches have been efficiently used to characterize vector and pathogen interactions: in *Drosophila melanogaster*, one of the most well studied genetic model organisms (McTaggart et al., 2015), and *Anopheles* spp. mosquitoes (Domingos et al., 2017). The characterization of tick organs response to infection using technologies such as transcriptomics, proteomics, and functional genomics improved current knowledge on tick–pathogen interactions and allowed the development of new strategies and/or the identification of targets for tick and disease control. Also, research has shown that during the long-lasting tick–pathogen co-evolution, microorganisms have developed important strategies to manipulate or modulate tick response to infection, without impairing tick survival, enhancing their capacity of infection, replication, and transmission guaranteeing the survival of both (de la Fuente et al., 2016; Šimo et al., 2017). In *Ixodes scapularis*, the effect of *Anaplasma phagocytophilum* infection on tissue-specific responses and on cellular pathways, such as apoptosis or stress response, which can be activated in a certain tissue in response to infection in order to contain parasite evasion and or improve immune response, have been extensively studied (Alberdi et al., 2015; Ayllon et al., 2015; Villar et al., 2015; Cabezas-Cruz et al., 2017). However, there is limited knowledge on tick vector responses to *Babesia* spp. infection. *Babesia* parasites invade several tick tissues including the midgut, salivary glands (SGs), and ovaries, affecting tick fitness. Some tick molecules such as calreticulin, a calcium binding protein, kunitz-type serine protease inhibitors, lachesin, vitellogenins, among others, have been identified as having a role on tick–*Babesia* interface (Zhou et al., 2006; Rachinsky et al., 2008; Antunes et al., 2017, 2018). Scarce information is available regarding the mechanisms used by *B. bigemina* to infect, develop, multiply, and survive inside the tick vector. Additionally, the impact of parasite infection at tick transcriptome and proteome levels, particularly on the molecular pathways affected by *B. bigemina*, is still to be investigated.

The overall objective of this study was to deepen the understanding on the complex *R. annulatus* response to *B. bigemina* infection. To conclude on mRNA and protein levels of *R. annulatus* in response to *B. bigemina* infection, the present research combined transcriptomics and proteomics analysis to obtain a sialotranscriptome and proteome by RNA sequencing (RNA-seq) and reversed phase liquid chromatography–mass spectrometry (RP-LC–MS/MS). Research focused on genes and proteins that were found differentially expressed or represented after infection. Six genes were further studied by RNAi-mediated gene silencing including genes related to apoptosis and stress response. This work represents the first report concerning the effect of *B. bigemina* infection on the sialotranscriptome and proteome of *R. annulatus*, and in the influence of the presence of the parasite on specific and crucial cellular pathways, constituting an important step further on the development of new measures for ticks and parasite control.

## Materials and Methods

### Ethics Statement

Animal experiments were conducted according with the “Guide for Care and Use of Laboratory Animals” of the institutions involved in the study, following protocols approved by the Committee on the Ethics of Animal Experiments and the principle of the three Rs, to replace, reduce, and refine the use of animals for scientific purposes.

### *Rhipicephalus annulatus* and *Babesia bigemina*

*R. annulatus* adult ticks were obtained from a laboratory colony free of tick-transmissible infections maintained at the Kimron Veterinary Institute, Israel. Six-month-old Friesian calves were tested for the presence of antibodies against *Babesia* spp. using an immunofluorescence assay, as described previously (Shkap et al., 2005), and were kept under strict tick-free conditions. To obtain *B. bigemina*-infected ticks, one calf was splenectomized and inoculated intravenously with 1 × 10^6^ *B. bigemina* (Moledet strain) cryopreserved parasites a fortnight later. Once the peak of parasitemia was reached, ticks were fed for 1 week inside cotton bags attached with non-toxic silicone glue GE Advantage Silicone Sealant (General Electric, New York, NY, United States) to a shaved area on the dorsal region of the animal. Similarly, a naïve calf was used to obtain uninfected ticks. Detached adult female ticks from the infected and naïve calf were maintained at 28°C and 80% humidity.

### Sialome

#### Sample Preparation and Probe-Based qPCR Detection of *Babesia bigemina*

A total of 30 uninfected and 30 *B. bigemina*-infected female ticks were used for tissue isolation. Ticks were washed twice in distilled water, once in 75% (v/v) ethanol, and one last wash in water. Ticks were dissected and the SGs extracted under a Motic SMZ-171B stereomicroscope (Motic Instruments Inc., Xiamen, China), using ice-cold phosphate buffer saline (PBS). Total RNA from 10 SGs obtained from each group was extracted using TriReagent (Sigma–Aldrich, St. Louis, MO, United States), according to the manufacturer’s instructions. RNA concentration and integrity were evaluated by analysis of rRNA band integrity using Agilent 2100 Bioanalyzer (Agilent Technologies Inc., Santa Clara, CA, United States). cDNA was synthesized from 100 ng of total RNA using the iScript cDNA Synthesis Kit (Bio-Rad, Hercules, CA, United States), following the manufacturer’s protocol. To determine the presence of *B. bigemina* in the SGs, probe-based qPCR reactions were carried out as described before for the detection of 18S rRNA of *B. bigemina* (Kim et al., 2007). Briefly, triplicate 20 μl reactions were prepared with 10 μl of Probe Xpert Fast Probe 2× Mastermix (GRISP Research Solutions, Porto, Portugal), 400 nM of each primer, 100 nM of probe, 1 μl of cDNA template, and nuclease-free water up to the final volume. The qPCR was carried out in a CFX Connect^TM^ Real-Time PCR Detection System thermal cycler (Bio-Rad, Hercules, CA, United States) with a thermal cycling profile of 10 min at 95°C, followed by 45 cycles of 20 s at 95°C and 1 min at 55°C. Negative controls were prepared with no template. Positive controls were prepared with *B. bigemina* Moledet strain purified DNA. A standard curve was constructed with fivefold serial dilutions of the positive control DNA to determine reaction efficiency. Data were analyzed using the Bio-Rad CFX Manager Software version 3.1 (Bio-Rad, Hercules, CA, United States). Samples with quantification cycle (Cq) values above 39 were considered negative for the presence of the pathogen. After *B. bigemina* infection confirmation, the remaining 20 SGs from infected or uninfected ticks were grouped in two pools, and the total RNA, for RNA-seq was extracted as described above.

#### RNA Sequencing and Analysis

Library preparation was performed using TruSeq RNA kit (Illumina, San Diego, CA, United States), following the manufacturer’s instructions. Shortly, prior to cDNA library construction magnetic beads with Oligo (dT) were used to enrich poly (A) mRNA from 1 μg of total-RNA. Purified mRNA was disrupted into short fragments. Two different fragmentation conditions were applied so that a “shorter” and a “longer” preparation were made for both the control (uninfected) and the *B. bigemina* infected RNA samples. Next, the purified mRNA was disrupted into short fragments, and double-stranded cDNA was immediately synthesized. cDNA was subjected to end-repair and adenylation, then connected with sequencing adapters. Suitable fragments, purified by size selection protocol with AMPure XP beads (Beckman Coulter, Pasadena, CA, United States), were selected as templates for PCR amplification. The final library sizes and qualities were evaluated by electrophoresis using an Agilent High Sensitivity DNA Kit (Agilent Technologies Inc.); fragment size range was between 287 and 296 bp for the short insert and 397 and 436 bp for the longer insert preparations. Subsequently, libraries were pooled and titrated using qPCR to obtain an accurate estimation of concentration. Cluster generation was performed in a Cluster Station (Illumina, San Diego, CA, United States) and the libraries sequenced using a GAIIx equipment (Illumina, San Diego, CA, United States), with a 2 × 100 cycle sequencing reads separated by a paired-end turnaround. Image analysis was performed using the HiSeq control Software version 1.8.4. (Illumina, San Diego, CA, United States) at Parque Científico de Madrid, Spain.

#### Assembly and Analysis of Transcripts

Reads were trimmed where the error probability was higher than 0.05. To get an optimal assembly, the sequence reads included were only those that the two members of the pair remained after filtering and trimming. Oases Software (Velvet, version 1.2.10) suitable for short paired-end reads assemblies (Schulz et al., 2012) was used selecting the mode of single assembly, i.e., not merged. For the two conditions, a kmer of 83 was selected for being close to the total length of the read (115 bp), avoiding misassembles. The Transcriptome Shotgun Assembly (TSA) project was submitted to the DDBJ/EMBL/GenBank under accession numbers GBJT00000000 and GBJS00000000 for uninfected and infected ticks, respectively. Functional annotation of each transcript was conducted based on the Basic Local Alignment Search Tool (BLAST^1^) and the results inferred by similarity to UniProt database^2^ reference proteins. The minimum similarity threshold required for annotating a transcript was a BLAST *E*-value < 10^−10^. A total of 34,095 reference proteins, considered to be representative on the UniRef90 clusters belonging to the taxonomic node Chelicerata, were downloaded from the UniProt database (January 2015). Chelicerata is eight levels above the *Rhipicentor reticulatus* taxon (Madder et al., 2014). Each selected protein belonged only to one cluster, showing 90% similarity with all members of that cluster. A protein-centred analysis of the differential expression was performed for the *de novo* transcriptome comparison. For the UniGene cluster formation, in each sample, for a set of transcripts annotated with the same UniProt, the longer nucleotide sequence was chosen to be the representative of each UniGene. Functional data were curated using Blast2GO platform version 4.0.7 available at https://www.blast2go.com (Conesa et al., 2005; Götz et al., 2008). Manual annotation was done with sequences retrieving no hits needed by compiling information from UniProt, RefSeq, GO, Panther, KEGG, Pfam, and NCBI databases. For the uninfected and infected condition comparison, transcripts were clustered by proteins. When two transcripts were annotated under the same UniProt they were included in the same cluster; and when the same protein cluster was present in both conditions, the protein cluster expression levels were compared. The software edgeR, for Empirical Analysis of Digital Gene Expression Data in R [Bioconductor version: Release (3.7)] (Robinson et al., 2010), was used to compare the mRNA expression levels detected in samples.

#### Transcriptomics Data Normalization and Validation

For transcriptomics data normalization, four reference genes were selected including *tubulin-beta-2B chain*, *elongation factor 1*, *cyclophilin*, and *transcription factor TFIID* (Robinson et al., 2010). Since no biological replicates were used, a biological coefficient of variation (BCV) was firstly selected to 0.4. Considering that if the BCV was perfectly approximated, the *p*-value for the four genes would have to be 1, the lowest *p*-value obtained for any of these four genes was selected. This corresponded to the *transcription factor TFIID* that had a *p* = 0.465 (for BCV = 0.4). All the genes with a *p*-value higher than 0.465 were selected as genes without differential expression to approximate the dispersion. The dispersion and the BCV were analyzed for this set of 2719 theoretically housekeeping genes. A BCV = 0.6 was obtained after approximating the dispersion of the housekeeping genes and this BCV value was used to select the differentially expressed genes in response to infection. For RNA-seq data validation, 10 paired SGs obtained individually from each uninfected and *B. bigemina*-infected *R. annulatus* female ticks (previously screened for infection), respectively, were used in triplicate. Supplementary Table S1 shows the primer sets used for qPCR and their respective final concentrations and annealing temperatures. Briefly, 10 μl triplicate reactions were prepared with 5 μl iTaq^TM^ Universal SYBR^®^ Green Supermix (Bio-Rad, Hercules, CA, United States), specific primer concentration, 1 μl of cDNA template, and nuclease-free water up to the final volume. The qPCR was carried out in a CFX Connect^TM^ Real-Time PCR Detection System thermal cycler (Bio-Rad, Hercules, CA, United States), with a thermal cycling profile of 10 min at 95°C, followed by 45 cycles of 15 s at 95°C and 45 s at specific-annealing temperature. Negative controls were prepared with no template. A standard curve was constructed with fivefold serial dilutions of *R. annulatus* cDNA to determine reaction efficiency. To ensure that only one amplicon was formed, a melting curve was performed at the end of every reaction (55–95°C; 0.5°C/s melt rates). To confirm correct fragment amplification, gel electrophoresis was performed, the fragments were sequenced by the chain termination method at StabVida (Lisbon, Portugal) and the resulting sequence was analyzed against the sequences available at the NCBI nucleotide database^3^. The CFX Manager^TM^ Software (Bio-Rad, Hercules, CA, United States) was used to analyze the gene expression data between conditions and reference gene validation was based on the geNorm algorithm (Vandesompele et al., 2002) and on the expression stability value M of each reference gene (*M* < 1).

### Proteome

#### Sample Preparation

A total of 16 uninfected and 16 *B. bigemina*-infected engorged female ticks obtained as described in the section “*Rhipicephalus annulatus* and *Babesia bigemina*” were equally divided into two different biological samples for each group. Ticks were dissected, cuticle removed, and washed in 10 mM PBS to eliminate the maximum host blood possible. Internal tick tissues were homogenized with a glass homogenizer (20 strokes) in lysis buffer [0.25 M de sucrose, 10 mM Tris–HCl pH 7.5, 1 mM MgCl_2_, 0.7% DDM, 0.5% ASB detergent, supplemented with Complete protease inhibitor cocktail (Roche, Basel, Switzerland)] in a ratio of 1 ml of buffer *per* gram of sample. Samples were sonicated for 1 min in an ultrasonic cooled bath (JP Selecta, Barcelona, Spain) followed by 10 s of vortex. After three cycles of sonication-vortex, the homogenates were centrifuged at 200 × *g* for 5 min at 4°C, to remove cellular debris. The supernatants were then collected and protein concentration was determined using the Bicinchoninic Acid (BCA) Assay (Thermo Scientific, San Jose, CA, United States), according to the manufacturer’s instructions.

#### Proteomics

One-hundred and fifty grams of protein extracts *per* group were on-gel concentrated by SDS–PAGE and trypsin digested as described previously (Villar et al., 2014). The desalted protein digest was re-suspended in 0.1% formic acid and analyzed by RP-LC–MS/MS technique using an Easy-nLC II system coupled to an ion trap LTQ mass spectrometer (Thermo Scientific, San Jose, CA, United States). Peptides were concentrated (on-line) by RP chromatography using a 0.1 × 20 mm C18 RP precolumn (Thermo Scientific, San Jose, CA, United States), and then separated using a 0.075 × 100 mm C18 RP column (Thermo Scientific, San Jose, CA, United States) operating at 0.3 ml/min. Peptides were eluted using a 180 min gradient from 5 to 40% solvent B (Solvent A: 0.1% formic acid in water, solvent B: 0.1% formic acid in acetonitrile). Electrospray (ESI) ionization was performed using a Fused-silica PicoTip Emitter ID 10 mm (New Objective, Woburn, MA, United States) interface. Peptides were detected in survey scans from 400 to 1600 amu (1 mscan), followed by 15 data dependent MS/MS scans (Top 15), using an isolation width of two mass-to-charge ratio units, normalized collision energy of 35%, and dynamic exclusion applied during 30 s periods.

#### Data Analysis

The MS/MS raw files were searched against a compiled database containing all sequences from Ixodidae, *Bos taurus*, and *Babesia* (135,071, 32,156, and 23,215 Uniprot entries in September 2017, respectively)^4^ and with a database created from transcriptomics data (Holt et al., 2002; Evans et al., 2012), according to the Proteomics Informed by Transcriptomics (PIT) technique using the SEQUEST algorithm (Proteome Discoverer 1.4, Thermo Scientific, San Jose, CA, United States). The following constraints were used for the searches: tryptic cleavage after Arg and Lys, up to two missed cleavage sites, and tolerances of 1 Da for-precursor ions and 0.8 Da for MS/MS fragment ions, and the searches were performed allowing optional Met oxidation and Cys carbamidomethylation. A false discovery rate (FDR) < 0.05 was considered as a condition for successful peptide assignments and at least two peptides *per* protein in at least one sample analyzed were the necessary condition for protein identification (Supplementary Table S2). Two biological replicates were used for each of uninfected and *B. bigemina*-infected ticks. For the quantitative analysis of tick proteins, after discarding *Babesia* and host proteins, the total number of peptide-spectrum matches (PSMs) for each tick protein was normalized against the total number of PSMs in ticks and compared between uninfected and infected ticks by χ^2^-test statistics with Bonferroni correction in the IDEG6 Software^5^ (*p* = 0.05). Only proteins that showed no significant differences between replicates and significant differences between the two conditions in at least three of the uninfected *versus* infected pair comparisons were considered as differentially represented (Supplementary Table S3). The mass spectrometry proteomics data have been deposited at the PeptideAtlas repository^6^ with the dataset Please check with the correction made in line 478 is fine.identifier PASS01339^7^.

#### Gene and Protein Ontology Analysis

Gene ontology (GO) terms were assigned to differentially expressed genes and represented proteins with the Software Tool for Rapid Annotation of Proteins (STRAP) version 1.5 (Cardiovascular Proteomics Centre of Boston University School of Medicine at http://www.bumc.bu.edu/cardiovascularproteomics/cpctools/strap/). For the analysis, two categories of GO terms were evaluated that included the Biological Process (BP) and Molecular Function (MF). Chord diagrams were generated to display GO and the expression/representation of apoptotic and stress response-related UniProt IDs by using the GOplot R package in RStudio (Version 1.1.453) (Walter et al., 2015).

#### Proteomics Data Validation

Proteomics data validation was conducted by Western blot using two commercial mouse monoclonal antibodies targeting homologous amino acids sequences between tick and mouse and an in-house rabbit anti-subolesin polyclonal antibody (Supplementary Table S4). Ten engorged female ticks of each condition (uninfected and infected) were dissected, internal organs were rinsed in PBS and used to extract proteinaceous content using TriReagent (Sigma–Aldrich, St. Louis, MO, United States), according to the manufacturer’s instructions. Elution was performed in 1× PBS, supplemented with 1% sodium dodecyl sulfate (SDS) and 1% protease inhibitor cocktail (Amresco, Solon, OH, United States). Protein concentration was assessed by spectrophotometry using a NanoDrop ND-1000 (Thermo Fisher Scientific). Fifty micrograms of protein extracts from each condition were re-suspended in Laemmli buffer (Bio-Rad, Hercules, CA, United States) containing 5% (v/v) of 2-β-mercaptoethanol, separated on a 12.5% discontinuous SDS–PAGE gel and transferred overnight at a constant 25 V to a nitrocellulose membrane, with a pore size of 0.2 μm (Bio-Rad, Hercules, CA, United States), using the Mini Trans-Blot^®^ Electrophoretic Transfer Cell (Bio-Rad, Hercules, CA, United States). Membranes were stained with Ponceau S and blocked with 5% (w/v) nonfat dry milk (Bio-Rad, Hercules, CA, United States) in PBS containing 0.05% (v/v) Tween 20 (PBS-T) at room temperature (RT), for 1 h. In parallel, polyacrylamide gels were stained with BlueSafe (NZYTech, Lisbon, Portugal) for 1 h at RT. Membranes were washed with Tris-buffered saline complemented with 0.05% (v/v) Tween 20 (TBS-T) and incubated with the primary antibody diluted according to the manufacturer or literature (Supplementary Table S4), for 90 min at RT. After washing, membranes were incubated with a goat anti-mouse Polyvalent Immunoglobulins (G, A, M) – Alkaline Phosphate secondary antibody (1:3000; Sigma–Aldrich, St. Louis, MO, United States) for 1 h at RT, and afterward washed with TBS-T. The antigen–antibody complexes were detected using the alkaline phosphatase (AP) conjugate substrate kit (Bio-Rad, Hercules, CA, United States) and the protein band intensities were estimated using ImageJ Software (version 1.51K) (Schneider et al., 2012).

### RNA Interference Assay

#### Ticks and dsRNA Synthesis

For the RNAi assay, *R. annulatus* pathogen-free adult ticks were obtained from the laboratory colony established at Knipling-Bushland U.S. Livestock Insects Research Laboratory and Veterinary Pest Genomics Center, Texas, United States, and maintained on cattle at the tick rearing facilities at the University of Tamaulipas, Mexico, as described in the section “*Rhipicephalus annulatus* and *Babesia bigemina.*” For the RNAi-mediated gene-silencing assays, gene-specific double stranded (ds) RNA was synthesized using as template the sequences assembled in this study and sequences publicly available in GenBank. In particular, *Rhipicephalus pulchellus* TSA GACK01000273, GACK01006332, GACK01002259, and GACK01006752, *Amblyomma maculatum* transcript JO843858 and *R. microplus* transcript U49934. Fragments of interest were amplified using the iProof^TM^ High-Fidelity PCR Kit (Bio-Rad, Hercules, CA, United States), with specific primers containing T7 promoter (Supplementary Table S5) and previously synthesized cDNA. Briefly, PCR reactions of 50 μL were prepared with 1× iProof HF Buffer, 10 mM of dNTP mix, 0.5 μM of each primer, 1 mM of MgCl_2_, 0.02 U/μl proof DNA polymerase, and 1 μl of template cDNA. The cycling conditions were as follows: initial denaturation at 98°C for 3 min, 35 cycles of denaturation at 98°C for 10 s, annealing at specific temperature (Supplementary Table S5) for 30 s and extension at 72°C for 15 s; and a final extension at 72°C for 10 min. To confirm correct fragment amplification, gel electrophoresis was performed on a 0.5× TBE 1.2% (w/v) agarose gel, purified using the Zigmoclean^TM^ Gel DNA Recovery Kit (Zymo Research, Irvine, CA, United States), and the fragments sequenced at StabVida (Caparica, Portugal). The resulting sequence was compared against the sequences available at the NCBI nucleotide database^8^. After correct confirmation of the amplified sequences, dsRNA was synthesized using the MEGAscript RNAi Kit (Ambion, Austin, TX, United States) accordingly with instructions provided by the manufacturer, purified, and analyzed by spectrophotometry and agarose gel.

#### Tick Inoculation and Cattle Infestation

**FIGURE 1:**
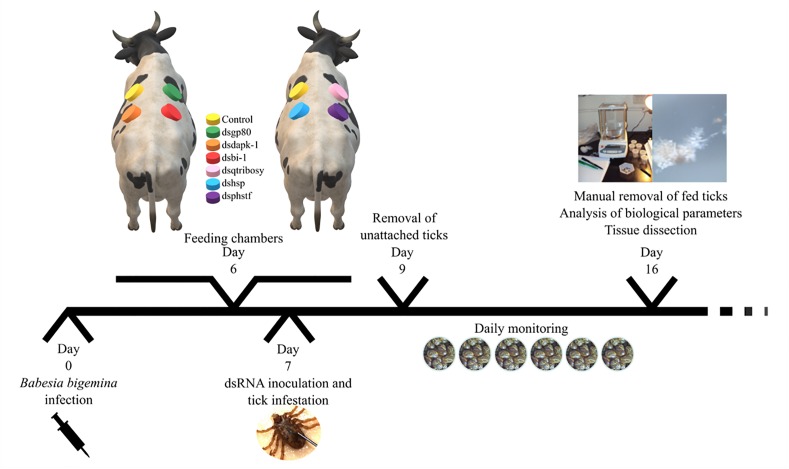
Experimental design of silencing assay. Two 6 months-old calves were inoculated intravenously with *B. bigemina*-infected blood. After 6 days, cotton cloth chambers were glued to the calf skin (one day before infestation). In day 7, thirty specific-dsRNA inoculated female ticks were added to its respective feeding chambers together with thirty males. Two days after infestation (day 9) unattached ticks were removed from the patches. Ticks were monitored daily and allowed to feed on the infected calves for 7 days. After this period, attached ticks were manually removed and stored in a humidity chamber until further use (analysis of biological parameters and tissue dissection).

Thirty pathogen-free *R. annulatus* unfed female ticks *per* group were injected with 1 × 10^11^ to 1 × 10^12^ molecules of specific dsRNA in the right spiracular plate, using a Hamilton syringe (Sigma) with a 1 in. × 33-gauge needle. The control group was injected with the same volume of elution buffer, similar to what has been described in other studies (Hussein et al., 2015; Ferrolho et al., 2017). While the use of unrelated dsRNA is now a well-established control in species with full genome annotation, the *R. annulatus* genome is not annotated thus increasing the possibility of off targets in silencing assays. After injection, ticks were kept in an acclimatized room at 22–25°C and 95% relative humidity, for 6 h before infestation. Two 6-month-old calves were inoculated intravenously with 2 ml of cryopreserved *B. bigemina*-infected blood (1 × 10^6^ infected erythrocytes) (field strain from Chiapas, Mexico). Cattle infection was confirmed by visual examination of blood smears. The cattle infestation was conducted as described in Figure 1. Ticks were monitored daily, at the second day unattached ticks were removed and the remaining were allowed to feed on the infected calves for 7 days. After this period, attached ticks were manually removed and stored in a humidity chamber until further use. SGs from 10 detached ticks *per* silenced-group were dissected and stored in RNA*later* (Ambion, Austin, TX, United States). The remaining 20 female ticks of each group were kept in individual pierced 1.5 ml microfuge tubes, under the laboratory conditions described above, for oviposition.

#### Efficiency of Gene Silencing, *B. bigemina* Infection Levels, and Tick Fitness

Gene silencing efficiency in SG was determined by comparing mRNA levels between specific dsRNA-injected and control groups by qPCR as described in the Section “Transcriptomics Data Normalization and Validation.” Quantitative PCR was carried out in both SG and ovaries for the 18S rRNA of *B. bigemina*, following a previously described protocol (Antunes et al., 2012), and the effect of gene silencing in infection was evaluated by a Student’s *t*-test (*p* = 0.05). Engorged female weight (EFW), egg mass weight (EMW), and egg production efficiency (EPE) were determined for each collected tick and analyzed between silenced and control groups by Student’s *t*-test with unequal variance (*p* > 0.05). The EPE was calculated according to the formula EPE = (EMW/EFW) × 100. Tick ability to feed was determined by the ratio of engorged female ticks collected from the feeding chambers to the total number of female ticks added initially, and statistically compared with the Chi-square test (*p* > 0.05) with the null hypothesis that tick mortality was independent of gene silencing.

## Results

### *R. annulatus* Female Sialotranscriptome: Assembly and Annotation

**FIGURE 2:**
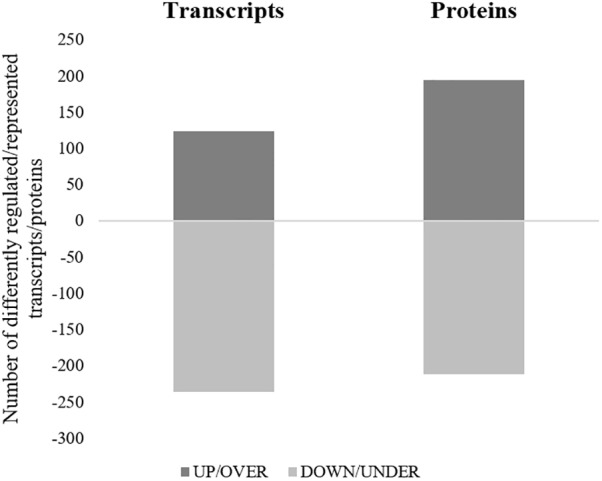
Representation of differentially regulated/represented transcripts/proteins of *R. annulatus* females in response to *B. bigemina* infection. For each approach, the number of transcripts (left column) or proteins (right column) differently regulated or represented are shown. Dark gray = up-regulated transcripts/over-represented proteins, Light gray = down-regulated transcripts/under-represented proteins.

**FIGURE 3:**
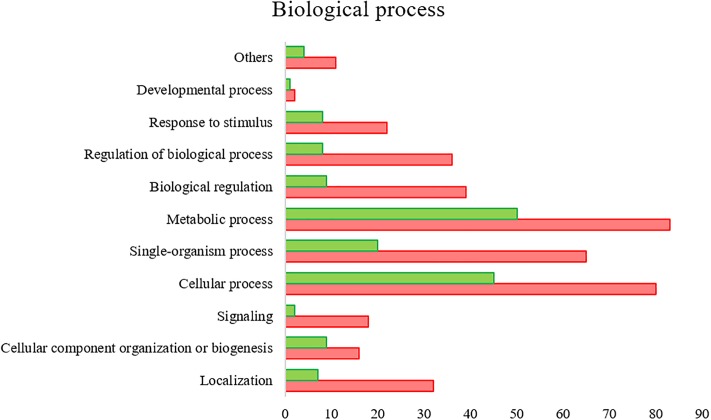
*R. annulatus* SGs transcriptional response to *B. bigemina* infection based on GO BP (level 2) of encoded proteins. Red bars represent down regulated genes, green bars represent up regulated genes with statistical significance (*p* < 0.05).

All the SG tested from *R. annulatus* female ticks fed on a *B. bigemina* infected calve were found to be positive for infection, while no infection was found on the SG of female ticks fed on the naïve calve. This enabled the production of a pool of infected SG and an uninfected SG pool to proceed with RNA-seq. With 40,573 988 high-quality paired-end reads, two transcriptomes were assembled, from the control and the infected samples. The assembly resulted in 33,379 putative transcripts in the control sample (33,118 with length < 200 bp) and 30,435 for the infected one (30,301 with length < 200 bp). After the first step of annotation, only the alignments (BLAST) with *e*-value minor than 10^−10^ were selected to infer functional annotation, resulting in 16,564 transcripts for the control and 15,037 for the infected sample. From these, 6823 unigenes with 1,374,576 ± 1,787,133 (Ave ±*SE*) estimated counts per protein were identified in the control population and 6475 unigenes with 1,182,677 ± 1,494,052 (Ave ±*SE*) in the infected group. Moreover, 884 unigenes were found to be exclusive to the infected samples while 1232 to the control samples (Supplementary Table S2). GO analysis of the obtained sialotranscriptome resulted in 69 sequences without BLAST hits, 877 sequences with BLAST hits without annotation, 473 with mapping without annotation, and 6286 sequences were functionally annotated. Supplementary Figures S1, S2 summarizes the functional annotation of the sialotranscriptome of *R. annulatus* female ticks. These multi-level charts show relevant functions from the general levels to the specific ones. The GO annotation highlights BPs such as single-organism cellular process, regulation of cellular process, gene expression or transport, and MF related to protein binding, hydrolase activity, or transferase activity. Using a BCV of 0.6, 360 unigenes were found to be differentially expressed (*p* < 0.05), whereas 123 were up regulated and 237 down regulated (Figure 2). Automated functional annotation was performed using Blast2GO and unigenes that did not retrieve GO terms were manually annotated. GO level 2 charts are shown in Figure 3, 4. The most represented BP were cellular and metabolic processes with 125 and 133 sequences, respectively. These categories include all processes carried out at the cellular level (e.g., apoptosis or homeostasis) and chemical reactions and pathways, including anabolism and catabolism, by which living organisms transform chemical substances. Biological regulation and regulation of BPs were represented by 48 and 44 sequences. Response to stimulus, including processes such as stress, immune, or redox state responses, was represented by 30 sequences. BP, such as growth or detoxification with a single representative, were included in the “other” BP category. Regarding the assignments of MF, the classes with higher representation, binding and catalytic activity, with 148 and 129 sequences, respectively, followed by transporter activity, with 26 sequences were found. With the exception of structural molecule activity, all the other classes contained more down regulated than up regulated transcripts.

**FIGURE 4:**
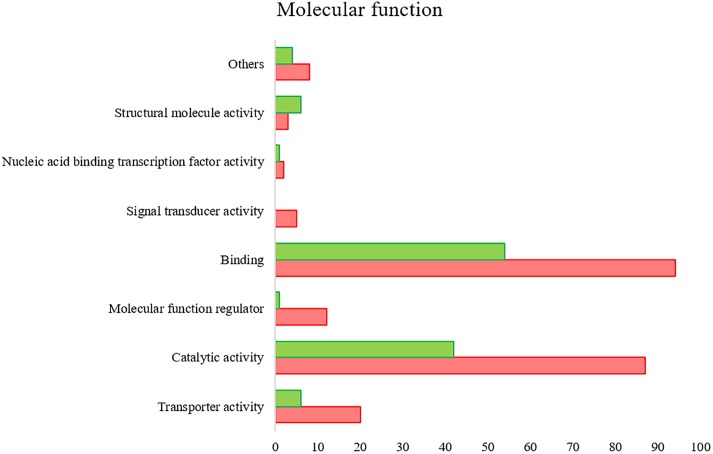
*R. annulatus* SGs transcriptional response to *B. bigemina* infection based on GO MF (level 2) of encoded proteins. Red bars represent down regulated genes, green bars represent up regulated genes with statistical significance (*p* < 0.05).

### Validation of RNA-Seq Results

To confirm differential gene expression, qPCR was performed on individual SGs. The levels of mRNA genes related to stress response encoding for the following proteins were analyzed: (1) small heat shock protein (HSP) II (UniProt ID:E0YPC0), (2) putative 60 kDa HSP (UniProt ID:L7M6W4), (3) putative heat shock hsp20 protein (UniProt ID:L7M7M1), (4) HSP 90 (UniProt ID:F1CGQ9), (5) Hsp70, putative (UniProt ID:L7M6A7), (6) putative selenoprotein k (UniProt ID:L7M5G4), (7) glutathione peroxidase (UniProt ID:Q2XW15), (8) thioredoxin peroxidase (UniProt ID:L7M1W2), (9) adenylate kinase isoenzyme 6 (UniProt ID:L7M323), (10) tumor rejection antigen-Gp96 (UniProt ID:B7QC85), (11) putative HSP (UniProt ID:L7MFS4), (12) serum amyloid A protein-like (UniProt ID:A6N9S3), (13) dual oxidase 1 (UniProt ID:B7PVC0), (14) putative HSP (UniProt ID:L7M4B9), and (15) putative heat shock transcription factor (PHSTF) (UniProt ID:L7MFL0). In addition, genes encoding for proteins related to apoptosis metabolic pathway were selected for validation of RNAseq by qPCR: (16) cytochrome c oxidase Subunit 1 (UniProt ID:A0A059VIA9), (17) putative death-associated protein kinase dapk-1 (DAPK-1) (UniProt ID:L7MKM3), (18) putative apoptosis inhibitor 5 (UniProt ID:L7LVN8), (19) bax inhibitor-1 related (BI-1) (UniProt ID:G3MPQ7), (20) putative apoptosis antagonizing transcription factor (UniProt ID:L7MAJ6), (21) mitochondrial ribosome small subunit component mediator of apoptosis dap3 (UniProt ID:L7M383) and (22) queuine trna-ribosyltransferase (QtRibosyl) (UniProt ID:L7M340), and (23) GP80 (UniProt ID:Q17174) related to proteolysis and lipid transport, respectively (Supplementary Figure S3). A moderate positive correlation between the mRNA levels by RNA-Seq and qPCR was obtained (Pearson’s correlation coefficient 0.6387, *p* = 0.001).

### Proteomics of the *R. annulatus* Female Ticks in Response to *B. bigemina* Infection

**FIGURE 5:**
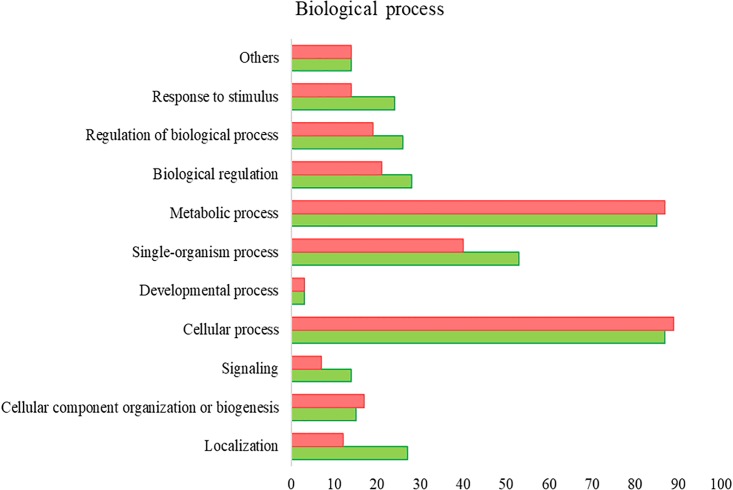
Gene ontology of the proteins from *R. annulatus* in response to *B. bigemina* infection. GO terms and its representation of BP (level 2). Green bars show over-represented proteins and red bars under-represented proteins with statistical significance (*p* < 0.05).

**FIGURE 6:**
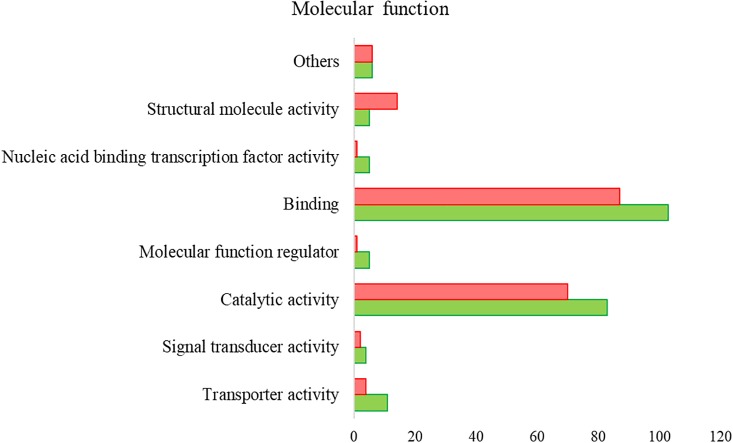
Gene ontology of the proteins from *R. annulatus* in response to *B. bigemina* infection. GO terms and its representation of MF (level 2). Green bars show over-represented proteins and red bars under-represented proteins with statistical significance (*p* < 0.05).

Proteomics analysis identified a total of 4,594 proteins in *R. annulatus* female ticks (Supplementary Table S2), where 80.08% (*n* = 3,679) were peptide sequences related to ticks while the remaining were mainly associated with the bovine host. Supplementary Figure S2 summarizes the functional annotation of the proteome of *R. annulatus* in multi-level charts. When comparing uninfected and *B. bigemina*-infected samples, 406 proteins were found differently represented (*p* < 0.05) and those were annotated using Blast2GO and UniProt-related databases (Supplementary Table S3). The proteomics data showed that *Babesia* infection leads to a higher number of under-represented proteins (*n* = 212), in comparison to over-represented (*n* = 194) (Figure 2). GO analysis contributed for the annotation of 89.41% differentially represented proteins (*n* = 363), while only 43 proteins were classified as “Unknown” due to the absence of annotation. The level 2 GO terms for BP and MF are shown in Figure 5, 6. The most represented GO terms were cellular, metabolic, and single-organism processes (BP, Figure 5), binding, catalytic, and nucleic acid-binding transcription factor activity (MF, Figure 6). Focusing on BPs identified herein, only cellular component organization or biogenesis, and cellular and metabolic processes, showed a higher number of over-represented proteins than under-represented. Regarding the MFs, with the exception of structural molecule activity, all were found to be under-represented.

### Validation of Proteomics by Western Blot

For the validation of proteomics results, three antibodies against apoptotic and stress-related proteins, Ran, clathrin, and subolesin (4D8), were used for western blot analysis using proteinaceous extracts of fed-uninfected and fed-*Babesia*-infected ticks (Supplementary Table S4). The results corroborated proteomics data, showing that in response to *B. bigemina* infection Ran protein (UniProt ID: B7QIB6) is over-represented (Proteomics fold change = +∞; band intensity: NI = non-determinated, *I* = 5,849,430) while Clathrin (UniProt ID: B7PUK8) was down-regulated (Proteomics fold change = −3.7; band intensity: NI = 11,493.492, *I* = 5,064.865). The 4D8 protein was found to be over-represented in *Babesia-*infected ticks in comparison to the uninfected ticks (Proteomics fold change = +∞; band intensity: NI = 5,8119.247, *I* = 11,2021.586) (Supplementary Figure S4).

### Stress Response and Apoptosis: A System Biology Approach

**FIGURE 7:**
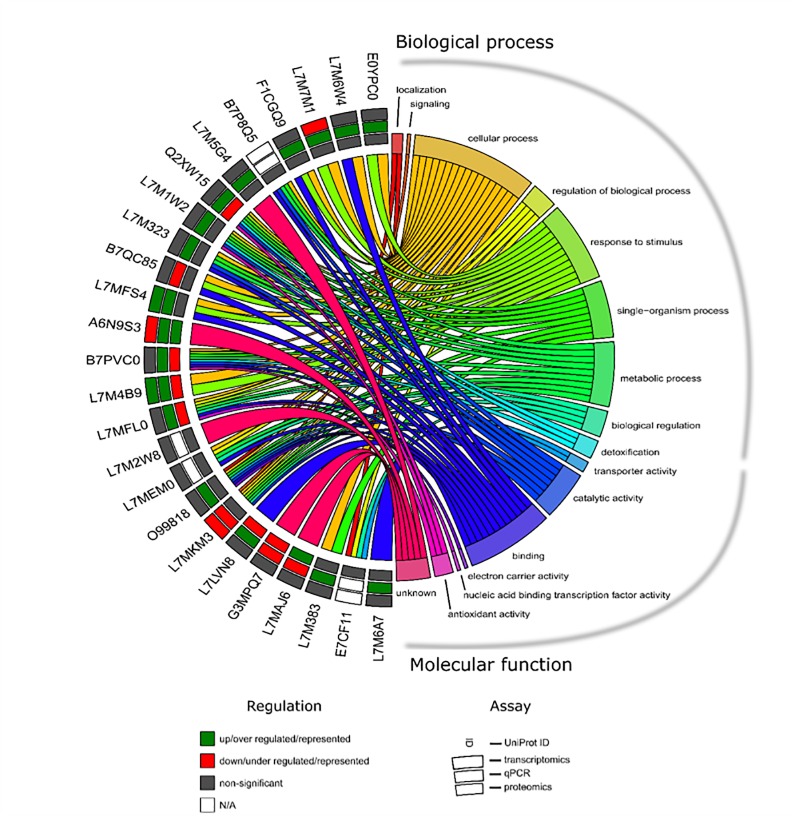
Chord diagram showing the apoptotic and stress response-related UniProt’s and its expression/representation as well as GO identified in *R. annulatus* in response to *Babesia* infection. Each target in each molecular approach is shown on the left, and the GO annotation is shown on the right including “BP” and “MF.” Outer annulus to inner annulus: transcriptomic, qPCR, and proteomic results. Green square: up/over expressed/represented; red square: down/under expressed/represented; gray square: non-significant (*p* > 0.05) and white square: not applicable.

All apoptotic and stress response-related molecules from transcriptomics and proteomics results were selected to further generate a chord diagram that displays the modulation of gene expression or protein production and its function (Figure 7). Eighteen and seven UniProt ID’s were found to be related to stress response and apoptosis, respectively. GO clusters such as regulation of BPs, response to stimulus, biological regulation, detoxification, and antioxidant activity were intertwined with these two pathways. Consistent and significant results between transcriptomics and qPCR approaches allowed the identification of three molecules, of which two were up-regulated HSP and putative heat shock-related protein (UniProt IDs: L7MFS4 and L7M4B9, respectively) and DAPK-1 was found down-regulated (UniProt ID: L7MKM3). From this dataset, only two targets were identified in both transcriptomics and proteomics databases, a putative heat shock-related protein (UniProt ID: L7M4B9) and serum amyloid A protein-like (UniProt ID: A6N9S3), with specific modulation of their expression and representation. Four UniProt IDs demonstrated an opposite correlation between cellular protein levels and mRNA abundance, glutathione peroxidase putative heat shock-related protein, PHSTF, and putative apoptosis inhibitor 5 (UniProt IDs: Q2XW15, L7M4B9, L7MFL0, and L7LVN8, respectively).

### Selection of Genes for RNA Interference

To gain insight into the functional role of identified tick vector genes/proteins in response to *Babesia* infection, targets for RNAi were selected based on their role in highly represented BPs such as apoptosis and stress response, and differential mRNA/protein levels in response to infection (Table 1). Two genes encoding for stress response-related proteins were selected (UniProt ID: L7MFS4 and L7MFL0), identified as being more expressed/represented in infected ticks. The first is a member of the HSP90 family, which is described as being able to regulate a specific subset of cellular signaling proteins that have been implicated in disease processes, particularly in ticks where they have been shown to have a role in virus replication (Weisheit et al., 2015). Transcriptional activation of heat shock genes relies on specific regulators such as the HSTF. Subsequently, a reduction of expression of such regulators may have a higher impact on activation of cellular stress response. Evidence of apoptosis manipulation in tick host cells by infecting pathogens has been demonstrated previously (Ayllon et al., 2015; Alberdi et al., 2016), which lead to the parallel selection of DAPK-1 and BI-1 genes (UniProt ID: L7MKM3 and G3MPQ7). The first, a pro-apoptotic kinase initially identified in vertebrate models, is involved in autophagy and tumor suppression (Inbal et al., 2002) and correspondent mRNA levels were found to be down regulated. The second, with an anti-apoptotic role (Lisak et al., 2015), appears to be under-represented in the current study. In the invertebrates *Drosophila* and *C. elegans* the role of DAPK-1 and related kinases has been briefly addressed revealing additional functions (Chuang and Chisholm, 2014). In ticks no functional studies have been performed so far with these two targets. The protein GP80 (UniProt ID: Q17174) is acknowledged to have a role in lipid transport and vitellogenesis (Tellam et al., 2002) thus correspondent reduction in mRNA levels is expected to deeply affect tick physiological parameters. QtRibosyl (UniProt ID: L7M340) is implicated in transfer RNAs (tRNAs) hypermodification, which in its turn are central players in nuclei acid translation (Lorenz et al., 2017). Particularly, queuine or Q-tRNA participate in many cellular functions, such as cell proliferation inhibition and stress (Vinayak and Pathak, 2009). In ticks, information of the impact of *QtRibosyl* depletion in tick physiological parameters, as well as in pathogen invasion, is still missing.

**Table 1:** Targets selected for RNAi assays, BPs in which are involved, and their representation on transcriptomics and proteomics analysis.

Gene	Encoding protein (Uniprot ID)	Biological process	mRNA (fold-change) infected/uninfected		Protein infected/uninfected
			RNA seq	qPCR	
*gp80*	Q17174	Lipid transport	−7.0^∗^	−7.67^∗^	
*Death-associated protein kinase-1*	L7MKM3	Apoptotic process	−3.31^∗^	−3.35^∗^	
*Bax inhibitor 1*	G3MPQ7	Apoptotic process	0.231	−1.724	−∞
*Heat shock protein*	L7MFS4	Stress response	3.25^∗^	3.027^∗^	
*Heat shock transcription factor*	L7MFLO	Stress response	0.07	1.293	+∞
*Queuine trna-ribosyltransferase*	L7M340	tRNA modification	0.15	4.18^∗^	−∞

*Red color indicates a significant down regulation or representation of the genes/proteins, the green color indicates a significant up regulation or representation of the genes/proteins while the gray color corresponds to non-significant regulation of the genes/proteins. ^∗^p < 0.05.*

### Functional Studies

#### Assessment of Gene Silencing and *B. bigemina* Infection

The results of gene knockdown efficiency in SG and its effect on *B. bigemina* infection in both SG and ovaries are shown in Table 2. Silencing was achieved for target genes *gp80* (98.6%), *bi-1* (92%), *hsp* (88.5%), and *QtRibosyl* (71.4%), as opposed to *phstf.* Despite testing different conditions, levels of *dapk-1* mRNA were not detected on the ds*dapk-1* inoculated ticks, suggesting an efficient knockdown. Silencing of *gp80*, *dapk-1*, and *bi-1* lead to a significant increase of infection levels in tick SG (*p* < 0.05) with a ratio of 2.29, 19.75, and 4.39, respectively. Knockdown of the remaining targets resulted in a non-significant decrease of infection levels of SG in comparison to the control group. Regarding the ovaries, a significant reduction of *B. bigemina* levels (*p* < 0.05) (ratio 0.16) was observed in response to *gp80* knockdown and in the remaining groups a non-significant increase was detected.

**Table 2:** Efficiency of gene knockdown by RNAi and its influence on *B. bigemina* infection levels in *R. annulatus* female ticks.

	Group	Gene silencing (%)	*B. bigemina* levels SG (Ave ± *SD*)	Test/Ct (Ave)	*B. bigemina* levels Ovaries (Ave ± *SD*)	Test/Ct (Ave)
Calf 1	*gp80*	∼98.6	4.74e^−04^ ± 1.53e^−04^	2.29^∗^	8.34e^−06^ ± 7.39e^−06^	0.16^∗^
	*Death-associated protein kinase-1*	^a^	4.09e^−03^ ± 4.23e^−03^	19.75^∗^	2.26e^−04^ ± 2.29e^−04^	4.18
	*Bax inhibitor 1 related*	∼92	9.07e^−03^ ± 9.91e^−04^	4.39	1.06e^−04^ ± 7.02e^−05^	1.96
	Control	–	2.07e^−04^ ± 1.23e^−03^	–	5.40e^−05^ ± 3.83e^−05^	–
Calf 2	*Heat shock protein*	∼88.5	1.80e^−03^ ± 2.16e^−03^	0.67	7.27e^−05^ ± 4.53e^−05^	2.46
	*Heat shock transcription factor*	ND	–	–	–	–
	*Queuine trna-ribosyltransferase*	∼71.4	1.86e^−03^ ± 2.35e^−03^	0.69	4.91e^−05^ ± 2.89e^−05^	1.67
	Control	–	2.70e^−03^ ± 3.88e^−03^	–	2.96e^−05^ ± 1.79e^−05^	–

*Thirty female R. annulatus ticks per group were injected with specific dsRNA or elution buffer as control. Ticks were allowed to feed in separated patches on calves experimentally infected with B. bigemina. Gene knockdown was analyzed by qPCR in 10 individual SGs per group by comparing mRNA levels between specific dsRNA-injected and control ticks using the CFX Manager^TM^ Software by means of the Pfaff method, ^∗^p < 0.05. The B. bigemina infection levels were determined in tick SG and ovaries by qPCR of the pathogen 18S rRNA gene and normalized against tick 16S rRNA using the ddCq method (2^−Cq^_target_^−Cq^_control_). Infection rate was calculated by the ratio of silenced per control groups. The mRNA levels and B. bigemina infection in ticks were compared between specific dsRNA injected and control ticks by a Student’s t-test (^∗^p < 0.05). ds, double-stranded; ND, not demonstrated; a, mRNA levels not detected in the silenced group.*

#### Biological and Reproductive Parameters of Tick After RNAi

Biological and reproductive parameters of *R. annulatus* female ticks injected with specific dsRNA before feeding are shown in Table 3. In all cases, the results obtained demonstrate that dsRNA inoculation did not affect the tick feeding ability (Chi-square, *p* > 0.05). Knockdown of *gp80*, *dapk-1*, *bi-1*, and *QtRibosyl* resulted in significantly lower female weights after feeding, in comparison to their respective controls (*p* < 0.05), and also in lower weight of laid eggs (*p* < 0.05). Females belonging to the *gp80*, *dapk-*1, and *QtRibosyl* silenced groups showed a significant (*p* < 0.05) reduction in EPE in comparison with their respective controls, which was not observed in the bi-1 silenced group. Four fed females belonging to the *QtRibosyl* silenced group were not able to lay eggs. *phstf* dsRNA inoculation resulted in lower female weight, egg mass, and EPE, but no statistical analysis was performed since silencing was not demonstrated.

**Table 3:** Evaluation of biological and reproductive parameters of *R. annulatus* female ticks injected with specific-dsRNA.

	Group	*n*	Attach (%)	EFW (mg Ave ± SD)	*n*	EMW (mg Ave ± SD)	EPE (Ave ± SD)
Calf 1	*gp80*	20	66.7	186 ± 56^∗^	10	64 ± 21^∗^	40.967 ± 7.280^∗^
	*death-associated protein kinase*	17	56.7	144 ± 61^∗^	7	52 ± 24^∗^	37.330 ± 3.882^∗^
	*Bax inhibitor 1 related*	18	60.0	200 ± 74^∗^	8	71 ± 30^∗^	47.622 ± 9.297
	Control	22	73.3	233 ± 35	12	113 ± 20	48.588 ± 3.735
Calf 2	*Heat shock protein*	19	63.3	226 ± 41	9	115 ± 29	50.763 ± 6.237
	*Heat shock transcription factor*	17	56.7	216 ± 41^a^	7	76 ± 10^a^	39.719 ± 3.335^a^
	*Queuine trna-ribosyltransferase*	20	66.7	149 ± 56^∗^	10	59 ± 25^∗b^	21.705 ± 19.982^∗^
	Control	20	66.7	242 ± 45	10	124 ± 28	50.015 ± 2.881

*Thirty R. annulatus female ticks per group were injected with dsRNA or elution buffer as control. Ticks were allowed to feed in separated patches on calves experimentally infected with B. bigemina. All attached ticks were collected after 7 days of feeding, weighed, and held in an acclimatized room. Ten ticks per group were randomly selected and SG dissected. The remaining were allowed to lay eggs. Tick ability to feed was evaluated as the ratio of removed ticks to the total number of ticks placed on the lamb using Chi-square test (^∗^p < 0.05). Female tick weight after feeding, EMW, and EPE were compared between dsRNA and unrelated dsRNA ticks by Student’s t-test. The EPE was calculated according to the formula EPE = (EMW/EFW) × 100. N, number of samples used in the statistical analysis of the subsequent parameters; EFW, engorged female weight; EMW, egg mass weight; EPE, egg production efficiency; ^∗^, statistical difference when compared to the control group. ^a^No statistical analysis was performed due to no gene knockdown. ^b^Four females didn’t laid eggs.*

## Discussion

Phylogenetic studies on certain pathogens and their tick vectors revealed a deep-rooted co-evolutionary relationship (de la Fuente et al., 2010; Antunes et al., 2017). The origin of piroplasmids could be traced back over millions of years to the time when tick species emerged, suggesting that *Babesia* parasites co-evolved with early tick lineages and their vertebrate hosts (Silva et al., 2011). Some pathogens may have a modest impact upon their vector and/or host, while others greatly influence host fitness and may manipulate host gene expression to favor infection, dissemination, and transmission (Cen-Aguilar et al., 1998; Mercado-Curiel et al., 2011; Antunes et al., 2017, 2018; Cornejo et al., 2017). We have found in our previous studies that the presence of *Babesia* in ticks induces a transcriptional shift (Antunes et al., 2012), particularly in the SGs (Antunes et al., 2018). Herein, a systems biology approach was applied to gain a more refined understanding on the complex interplay between *B. bigemina* and its tick vector *R. annulatus*. Our results identified *dapk-1*, *bi-1*, *gp80*, and *QtRibosyl* genes, that have important roles in apoptosis and vitellogenesis, and demonstrated that *Babesia* infection fundamentally affects key processes in SG tick cells.

It has been well documented that apoptosis is a highly regulated form of cell death. This process ensures a cell or organ to respond adequately to stress, thus limiting the risk of a disease or an infection that may compromise the ability of an organism to survive. Since its discovery, apoptosis has been extensively studied and its molecular regulation is now well understood. One of the critical components of this cell death pathway is *dapk-1*, a pro-apoptotic kinase, which can also regulate autophagy. Typically, *dapk-1* has been studied in the context of its suppression in tumor growth and metastasis but additional roles emerged in last years (Farag and Roh, 2019). In this study, we have shown that exposure of ticks to *B. bigemina* significantly down regulated the transcription of *dapk-1* in tick SG cells. This might have a significant impact upon the survival and propagation of *Babesia* parasites in its vector host. Inhibition of apoptosis in ticks and tick cells toward infection and survival is a strategy previously described in *Anaplasma* infection (Ayllon et al., 2015; Alberdi et al., 2016). Furthermore, silencing *dapk-1* led to a substantial increase in *B. bigemina* infection in the SG cells of ticks that fed on an infected calf. After a tick takes a blood meal, the SGs enlarge, then degenerate, and become atrophied. Recent evidence has demonstrated that SG transformation into a vestigial state is due to caspase-1-mediated apoptosis, limiting the potential for pathogen transmission (Yu et al., 2017). It is therefore interesting to observe the ability of *B. bigemina* infection to selectively restrict a vital gene within the apoptotic pathway. By blocking the apoptosis process, the parasite might very likely survive and persist in the tissue and further facilitate dissemination of the infection throughout the tick, and subsequent transmission to another host. In the event of apoptosis occurring in SGs, the parasite might be eliminated, likely *via* its destruction following the engulfment of the apoptotic bodies it is occupying. The *dapk-1* silenced ticks had significant lower weight than the control ticks, after a blood meal which might be attributed to the increased virulence observed, as this knockdown was not a spurious consequence of elevated biting rates. Such impact in female ticks has been previously described after silencing apoptosis-related genes in *A. phagocytophilum* infection in the tick vector *I. scapularis* (Ayllon et al., 2013, 2015). To the best of our knowledge, our study is the first ever reported to identify *dapk-1* from a high-resolution multi-omic screen of any *Rhipicephalus* tick species infected with *Babesia* parasites. Further research will seek to elucidate the molecular mechanism underlying *dapk-1* suppression in these cells and its functional significance.

The BI-1-related protein (UniProt ID: G3MPQ7) belongs to the Bax inhibitory protein-like family, exhibiting a highly conserved BI-1 domain throughout eukaryotes and prokaryotes (Lisak et al., 2015). Sequence analyses showed a high homology to an *Amblyomma triste* putative growth hormone-induced protein (UniProt ID: A0A023GIS5) and a growth hormone-inducible transmembrane protein-like from *Rhipicephalus zambeziensis* (UniProt ID: A0A224YYC2), also known as transmembrane BAX inhibitor motif (TMBIM) containing protein 5 supporting its anti-apoptotic role (Reimers et al., 2007; Rojas-Rivera and Hetz, 2015). TMBIM5 is recognized to be the only member from the TMBIM family of proteins localized mainly in the inner mitochondrial membrane and directly involved in the outer mitochondrial membrane permeability. Down regulation of such a protein leads to the release of pro-apoptotic proteins from the mitochondria, whereas overexpression results in the stabilization of cytochrome c at the inner membrane (Oka et al., 2008). Herein, the *R. annulatus* putative ortholog protein was found to be down regulated in response to infection, suggesting that under invasion there is an induction of cell apoptosis. However, reinforcement of gene down regulation resulted in the increase of *B. bigemina* levels in the SGs. Interestingly, the expression of this gene was also found to be down regulated in many types of cancer suggesting that the functional impact of its regulation is not fully understood yet (Rojas-Rivera and Hetz, 2015). Our results point to interactions between *Babesia* parasites and the tick apoptosis-related molecules, which requires further investigation with a special reference to this particular metabolic pathway.

In ticks, vitellogenesis is the process of yolk formation with the material accumulating in the developing oocyte and characterized by the synthesis of vitellogenins (Vg) during the reproductive period (Coons et al., 1989). GP80 is a processed product from vitellogenin, encoded by one *Vg* gene (Thompson et al., 2007; Taheri et al., 2014), and for simplicity we will maintain, herein, the GP80 nomenclature. Multiple *Vgs* have been described in ticks (Boldbaatar et al., 2010; Rodriguez et al., 2016; Xavier et al., 2018). Sequence analysis demonstrate a high similarity of *gp80* to *Vg-1* (UniProt ID: A0A034WTV5). In this study, *B. bigemina* infection led to a significant down regulation of *gp80* transcription, which is in contrast to our previous study results (Antunes et al., 2012). A possible explanation for this discrepancy relies on the different tissues analyzed in the two studies. In the present one, only SGs were targeted, whereas Vgs are considered to be absent and are thought to be functionally replaced by the hemelipoglyco-carrier protein (CP) (Donohue et al., 2009). Encountering transcription of the *gp80* in this organ suggests that *Rhipicephalus* SGs possess genes highly similar to Vgs, sharing many molecular features to since similar results were attained in SGs of *Rhipicephalus bursa* (Antunes et al., 2018). Our data revealed a significant decline in engorged female *R. annulatus* weight, EMW, and EPE, when *gp80* was silenced, upon feeding on an infected calf in accordance to previous studies (Boldbaatar et al., 2010; Antunes et al., 2018). Keeping in mind that the silencing methodology is not tissue specific, our results suggest that a decreased expression of *Vgs* leads to a reduction in lipid transport and normal production of energy (ATP) provided by lipid disruption, leading to an abnormal or delayed development of ovaries and eggs. A consequent increase in *B. bigemina* infectivity in SG was observed by silencing *gp80* and a parallel decrease was detected in the ovaries. This parasite sustains transmission via both horizontal and vertical routes, through feeding and eggs production, respectively, which imply that a parasite-driven strategy to restrict vitellogenin synthesis is less likely. This data suggest that the host may redirect resources away from cell defense against pathogens toward processes with a more urgent demand such as egg production. Therefore, once facing lack of *Vgs* transcription, which will ultimately affect progeny, cells efforts will go to compensate this process, leaving SG cells more prone to infection and pathogen dissemination. However, the opposite scenario was observed in the ovaries, whereas a decrease of the levels of *B. bigemina* was detected suggesting that parasite transmission is hampered by the GP80 deficiency. A similar effect has been observed in *Laodelphax striatellus* whereas the transmission of a virus is facilitated by the expression of an insect-specific *Vg* (Huo et al., 2018). In fact, it seems that the process of vitellogenesis is critical for efficient pathogen transovarial transmission and is has been suggested that *Babesia* may directly interact with a Vg receptor (Boldbaatar et al., 2008; Hussein et al., 2019). Targeting vitellogenesis to control tick infestation is a very attractive approach, since the inhibition or disruption of such process may result in tick female mortality, decreased egg production, and viability and, ultimately, have an effect on pathogens with transovarial transmission such as *Babesia* spp.

Transfer RNAs are primarily involved in translation, functioning as adaptors from DNA to proteins (Pathak et al., 2007; Lorenz et al., 2017). One of the most important tRNA hypermodification is the replacement of guanosine by an analog, the nucleoside queuosine (Vinayak and Pathak, 2009), resulting in queuine-tRNA (Q-tRNA) and implicating a cascade of enzymatic reactions (Betat et al., 2010). This Q-tRNA have been shown to correlate with diverse cell processes including stress tolerance, cell proliferation, tumor growth, and protein translation (Fergus et al., 2015; Tuorto et al., 2018). QtRibosyl, herein designated as QtRibosyl, is an enzyme involved in the chemical reaction that results in queuosine base modification (Deshpande and Katze, 2001; Fergus et al., 2015). In the present study, the expression of *QtRibosyl* was found to be up regulated while protein levels were found to be down regulated in response to infection. This may be due to several biological factors such as the occurrence of post-transcriptional and post-translational modifications but also due to methodological constraints (Maier et al., 2009; Ayllon et al., 2015; Villar et al., 2015). Interestingly, the human ortholog enzyme gene (*Queuine tRNA-ribosyltransferase catalytic subunit 1-QTRT1*) has been found differentially regulated in some cancer tissues. It has been shown that, as a consequence, the Q-tRNA deficiency is associated with different neoplastic tissues and may even correlate with tumor grade (Vinayak and Pathak, 2009; Fergus et al., 2015). A high expression of such enzyme suggests that the cell needs to produce Q-tRNA either to promote the activity of antioxidant enzymes, secure protein folding, or to maintain high glycolytic rate (Pathak et al., 2008; Fergus et al., 2015; Tuorto et al., 2018), processes which have been demonstrated to occur in infected ticks (Villar et al., 2015; Cabezas-Cruz et al., 2017). Moreover, the present study demonstrated that efficient knockdown of *QtRibosyl* affected tick parameters and led to a marked reduction of female weight, egg mass, and EPE. These findings are clearly supporting the role of Q-tRNA in cell proliferation and glycolytic metabolism. A non-significant reduction of *Babesia* infection in tick SG was also observed, which can be related to a deficit on cellular ATP pools, which in its turn results in a shortage of nutrients essential for parasite multiplication (Trager, 1974).

Stimulation of a cell stress response by pathogens in ticks has been previously reported (Mulenga et al., 2003; Villar et al., 2010; Busby et al., 2012; Weisheit et al., 2015). Herein, two heat shock-related genes were targeted: the *hsp* which was found to be up regulated upon infection and the *phstf* that showed a similar expression in SG on both conditions. Knockdown of *hsp* did not significantly affected the studied parameters, although further studies centered in this biologically relevant pathway in ticks should be performed to deepen our understanding on tick–*Babesia* interface.

Our study has pinpointed interesting tick proteins interacting with *Babesia*. Still, due to its transovarial and transstadial transmission capacity, future studies focusing on early feeding time points, other tick stages, or other tissues, such as ovaries, are needed to fully understand the molecular dynamics at the tick–pathogen interface.

## Conclusion

For many intracellular parasites, the host cell is no longer an opponent but rather an assistant, evolving to efficiently and precisely manipulate their host. Accordingly, in ticks, *Anaplasma* bacteria are able to subordinate cell BPs such as apoptosis and glycogenesis. The present study shed some light regarding the *Rhipicephalus*–*Babesia* interface by making use of transcriptomics and proteomics allied to gene functional analysis. The results obtained point out a strong parasitic pressure over cellular functions, of which processes like apoptosis and stress response stand out. The genes *dapk-1*, *QtRibosyl*, and *gp80* were characterized with regard to their role on tick reproductive parameters and interaction with *Babesia* infection, and showed to be highly influential functional molecules. The importance of understanding the tick biology and tick–pathogen molecular interactions is of the outmost importance in the discovery of suitable vaccine candidates to control tick populations and tick-borne diseases.

## Data Availability

The datasets generated for this study can be found in DDBJ/EMBL/GenBank and PeptideAtlas repository, Accession numbers GBJT00000000, GBJS00000000, and identifier PASS01339, respectively.

## Author Contributions

SA, JdlF, and AD designed the study. VS, MM, and JMC were responsible for tick rearing. SA, JC, JF, MV, and JdlF performed transcriptomics and proteomics analyses. SA and JC performed qPCR assays and western blots. JMC, ND, SA, JC, and JF performed the RNA interference studies. SA, JC, JdlF, GS, and AD performed data analysis and wrote the manuscript. All authors edited and approved the final manuscript.

## Conflict of Interest Statement

The authors declare that the research was conducted in the absence of any commercial or financial relationships that could be construed as a potential conflict of interest.

## Footnotes

1 https://blast.ncbi.nlm.nih.gov/Blast.cgi

2 https://www.uniprot.org/

3 https://blast.ncbi.nlm.nih.gov/Blast.cgi

4 http://www.uniprot.org

5 http://compgen.bio.unipd.it/bioinfo/software/

6 http://www.peptideatlas.org/

7 http://www.peptideatlas.org/PASS/PASS01339

8 https://blast.ncbi.nlm.nih.gov/Blast.cgi
