# Supplementary material for: Transcriptome and Proteome Response of Rhipicephalus annulatus Tick Vector to Babesia bigemina Infection
Source: Front Physiol. 2019 Apr 2;10:318. doi: 10.3389/fphys.2019.00318 (PMC6454348; doi:10.3389/fphys.2019.00318)
Supplement: Supplementary file 6 [file Presentation_1.pdf]

Transcriptome and proteome response of *Rhipicephalus annulatus* tick vector to *Babesia bigemina* infection

Sandra Antunes[1*], Joana Couto[1], Joana Ferrolho[1], Gustavo Seron Sanches[1], José Octavio Merino Charrez[2], Ned de la Cruz-Hernández[2], Monica Mazuz[3] Margarita Villar[4], Varda Shkap[3], José de la Fuente[4,5], Ana Domingos[1]

[1]GHMT - Global Health and Tropical Medicine, Instituto de Higiene e Medicina Tropical - IHMT, Universidade Nova de Lisboa – UNL, Rua da Junqueira 100, 1349-008 Lisboa, Portugal.
[2]Facultad de Medicina Veterinaria y Zootecnia, Universidad Autónoma de Tamaulipas, Km. 5 carretera Victoria-Mante, CP 87000 Ciudad Victoria, Tamaulipas, Mexico.
[3]Kimron Veterinary Institute, P.O. Box 12, Bet Dagan, 50250, Israel.Instituto de.
[4] SaBio. Instituto de Investigación en Recursos Cinegéticos IREC, CSIC-UCLM-JCCM, Ronda de Toledo s/n, 13005 Ciudad Real, Spain.
[5]Department of Veterinary Pathobiology, Center for Veterinary Health Sciences, Oklahoma State University, Stillwater, OK 74078, USA.

*** Correspondence:**
Sandra Antunes
santunes@ihmt.unl.pt

Supplementary figures S1 to S4.

A

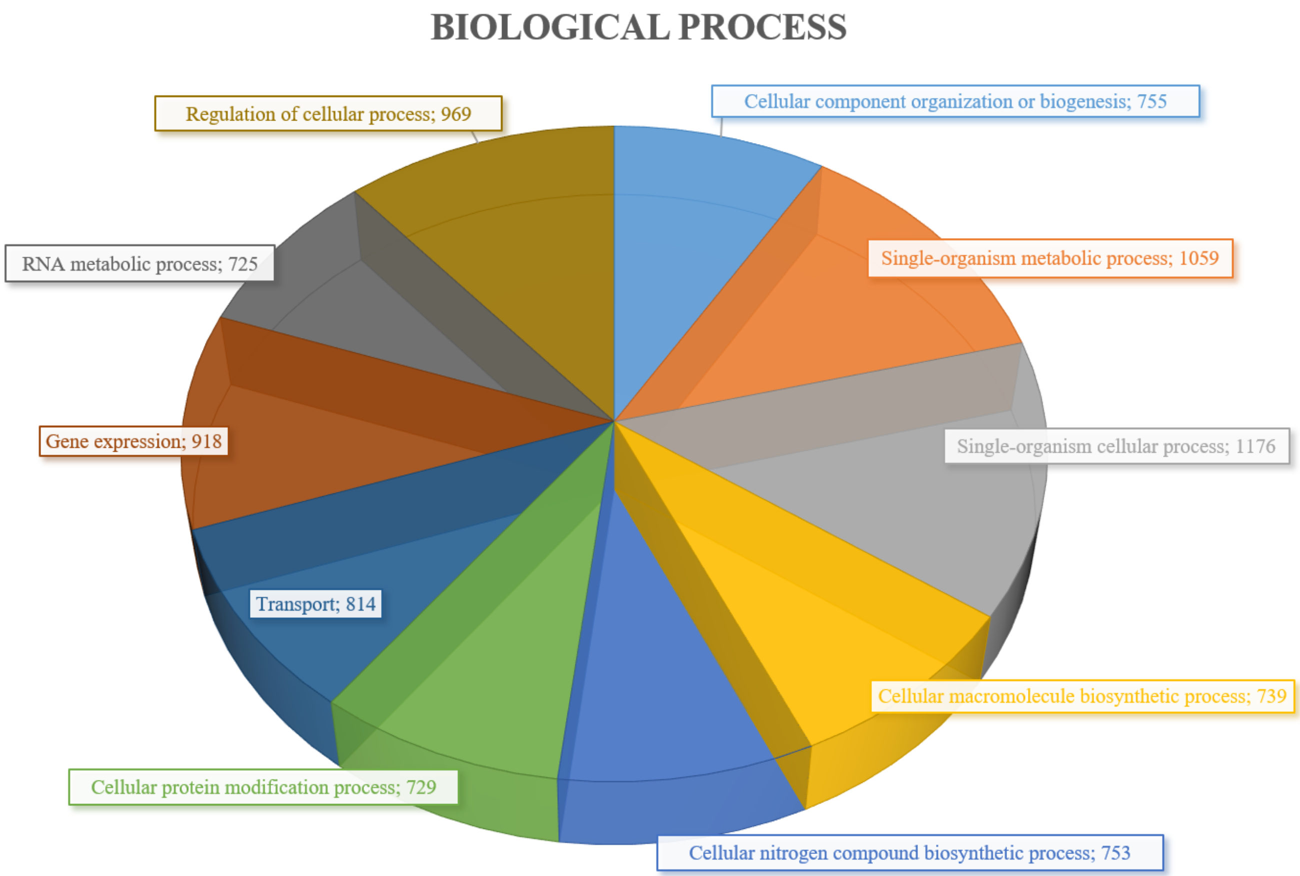

BIOLOGICAL PROCESS
Regulation of cellular process; 969
Cellular component organization or biogenesis; 755
Single-organism metabolic process; 1059
RNA metabolic process; 725
Single-organism cellular process; 1176
Gene expression; 918
Transport; 814
Cellular macromolecule biosynthetic process; 739
Cellular protein modification process; 729
Cellular nitrogen compound biosynthetic process; 753

B

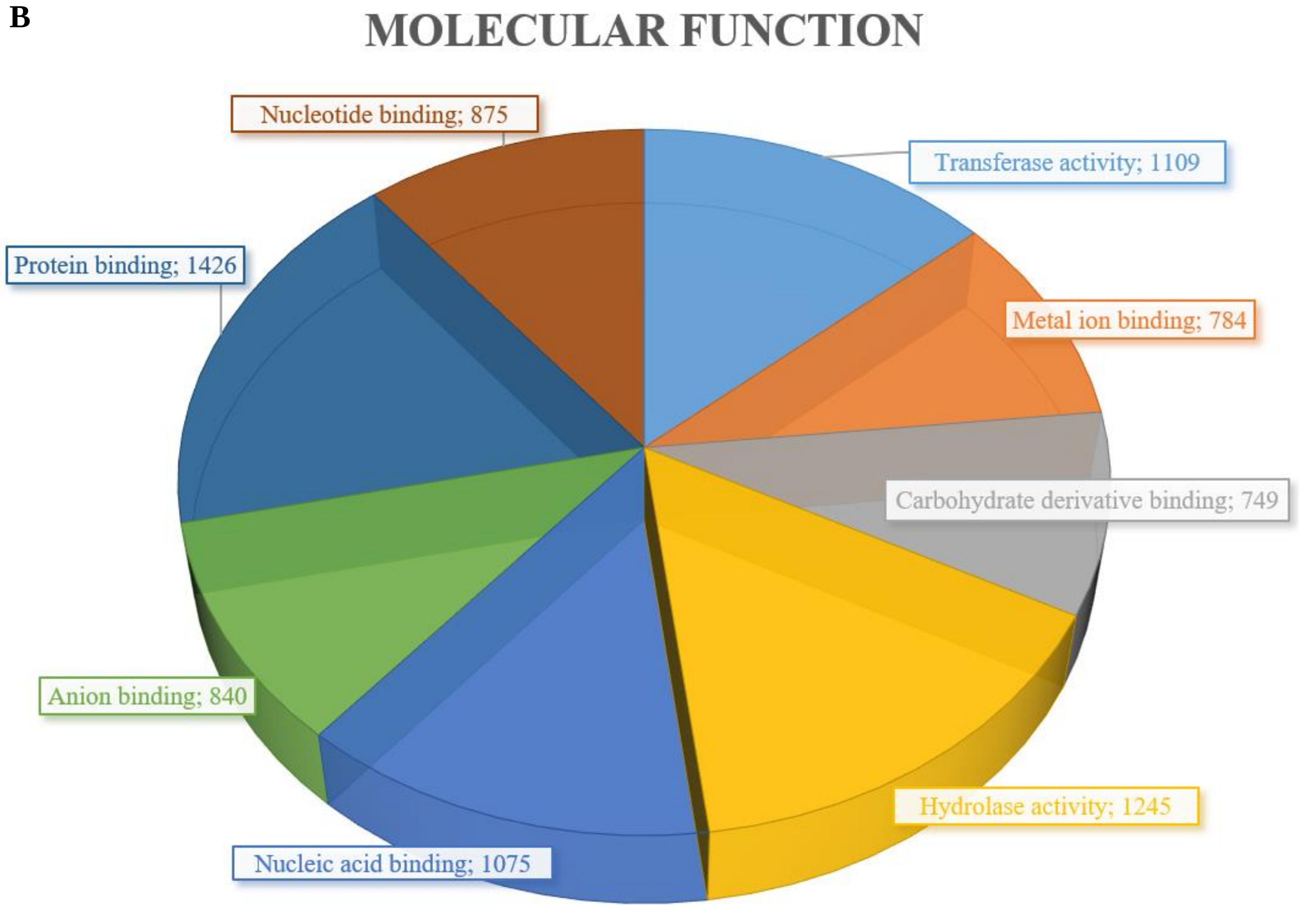

Figure S1**: Multi-level chart of biological processes(A) and molecular functions (B) of the sialotranscriptome of *R. annulatus* female ticks based in Gene Ontology.** For each slice a biological process or a molecular function is shown and its representation in number of transcripts.

A

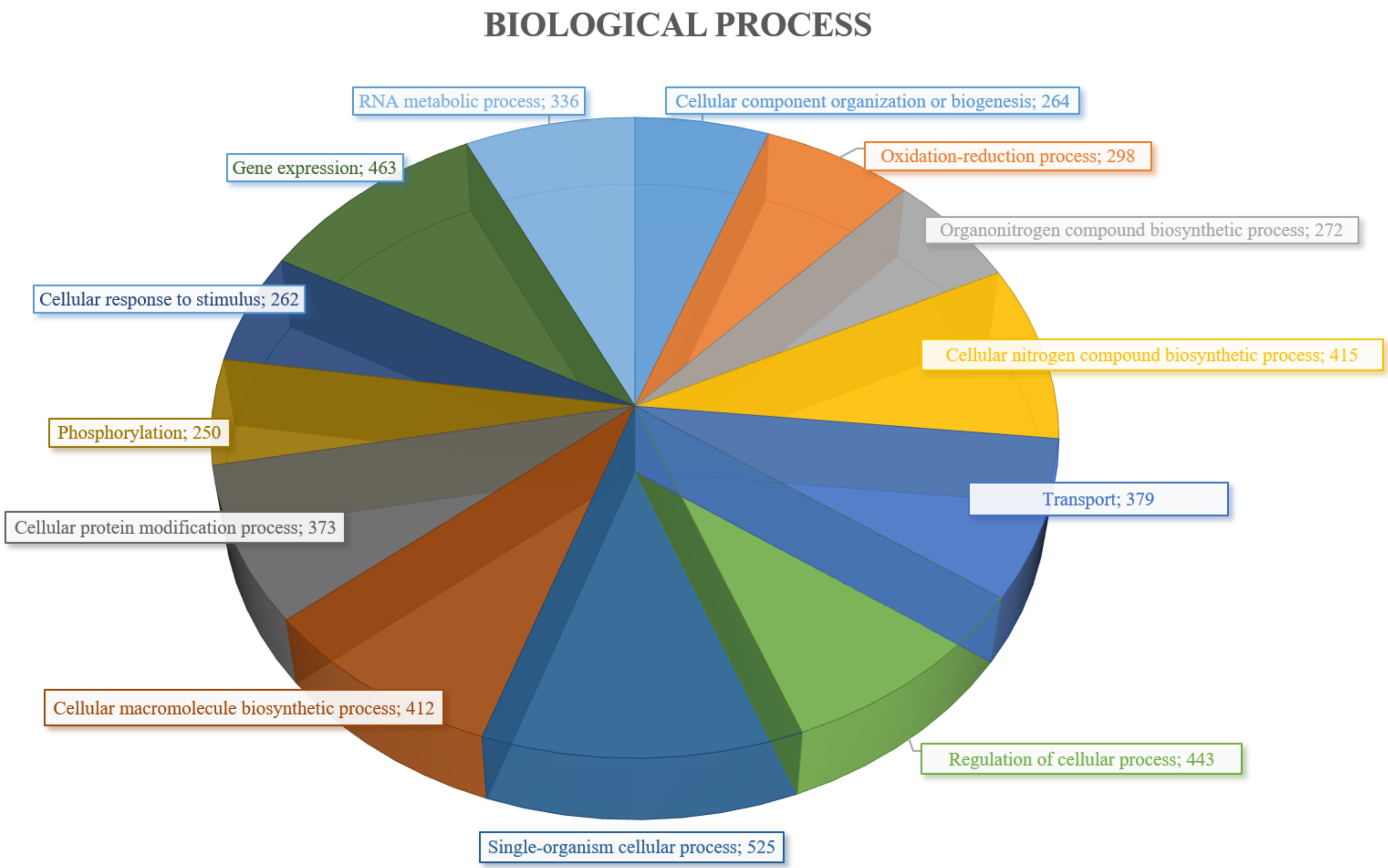
BIOLOGICAL PROCESS
RNA metabolic process; 336
Cellular component organization or biogenesis; 264
Oxidation-reduction process; 298
Gene expression; 463
Organonitrogen compound biosynthetic process; 272
Cellular response to stimulus; 262
Cellular nitrogen compound biosynthetic process; 415
Phosphorylation; 250
Transport; 379
Cellular protein modification process; 373
Cellular macromolecule biosynthetic process; 412
Regulation of cellular process; 443
Single-organism cellular process; 525

B

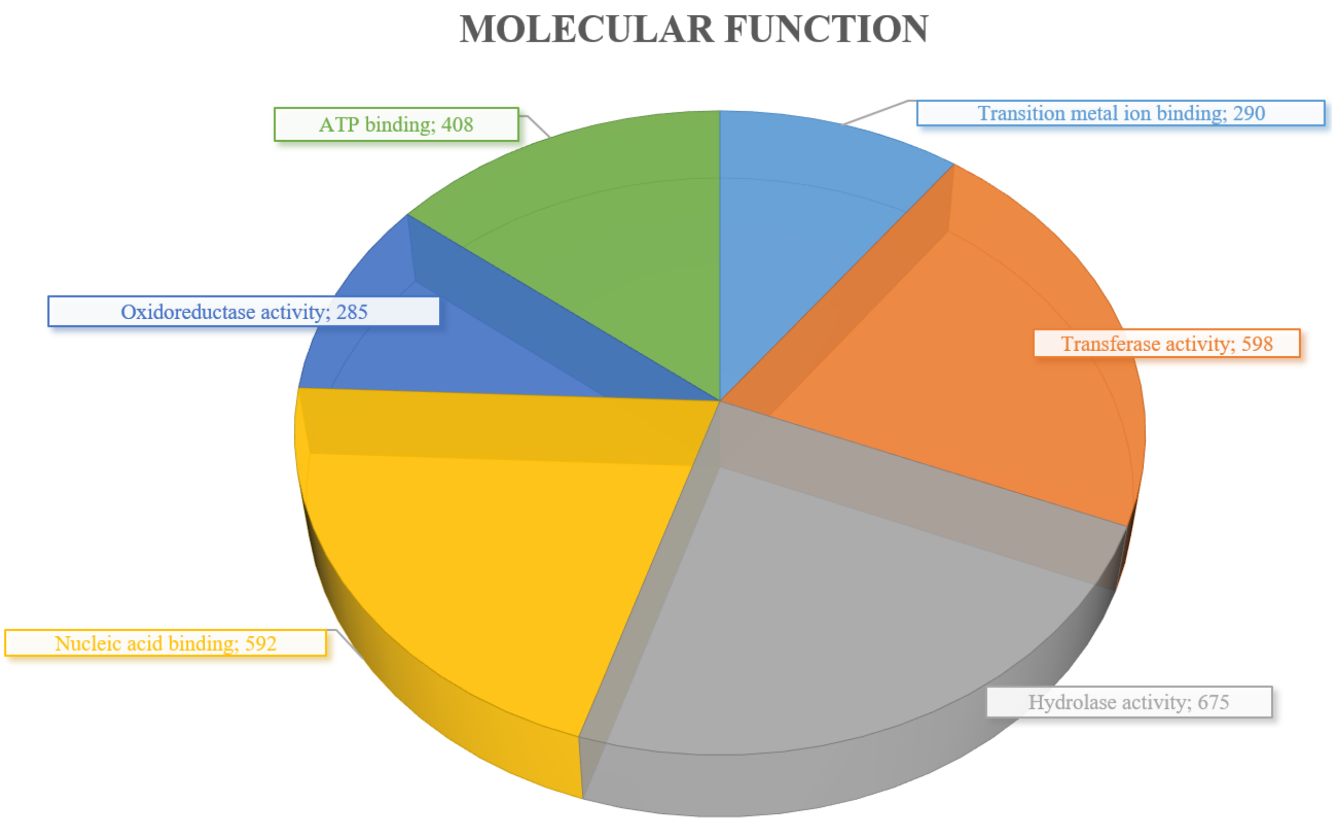

Figure S2: **Multi-level chart of biological processes(A) and molecular functions (B) of the proteome of *R. annulatus* female ticks based in Gene Ontology.** For each slice a biological process or a molecular function is shown and its representation in number of transcripts.

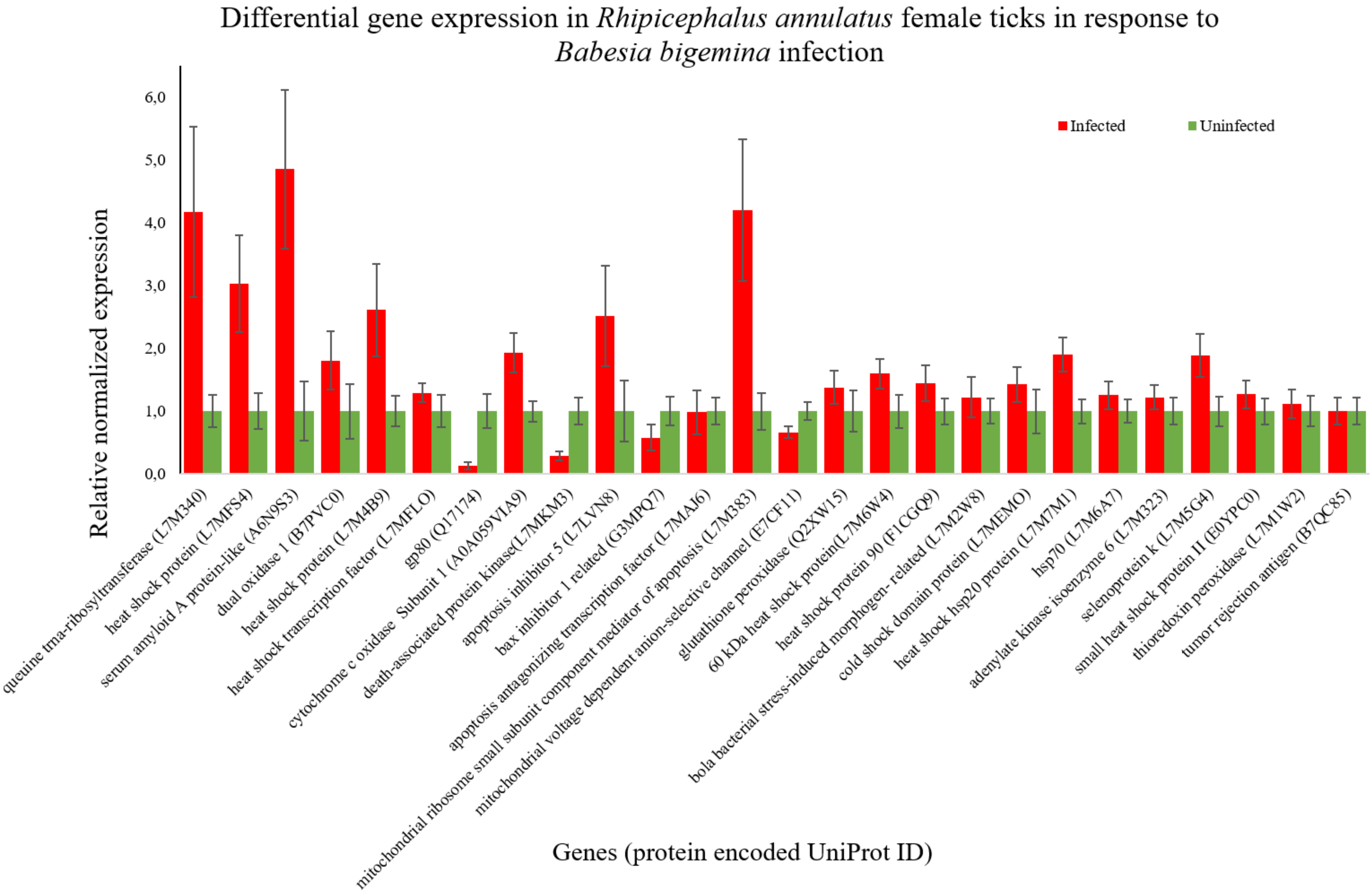

Figure S3**: Differentially gene expression of *R. annulatus* in response to *B. bigemina* infection evaluation by qPCR.** Red bars represent the *B. bigemina* infected SG and green bars represent the SG from uninfected *R. annulatus* female ticks. * $P < 0.05$

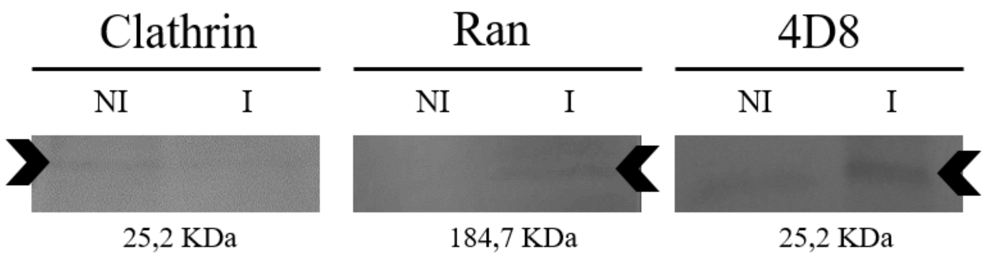

Figure S4: **Evaluation of differential representation of the proteins Clathrin, Ran and 4D8 in *R. annulatus* in response to *B. bigemina* infection by Western blot.** NI correspond to the uninfected sample; I correspond to the infected sample. The protein weight is referred below each panel.
